# Thermophysical properties of liquid Zr_52.5_Cu_17.9_Ni_14.6_Al_10_Ti_5_—prospects for bulk metallic glass manufacturing in space

**DOI:** 10.1038/s41526-019-0084-1

**Published:** 2019-10-25

**Authors:** M. Mohr, R. K. Wunderlich, D. C. Hofmann, H.-J. Fecht

**Affiliations:** 1 Institute of Functional Nanosystems, Ulm University, Albert-Einstein-Allee 47, 89081 Ulm, Germany; 2 Materials Development and Manufacturing Technology Group, NASA Jet Propulsion Laboratory /California Institute of Technology, Pasadena, CA USA

**Keywords:** Materials science, Statistical physics, thermodynamics and nonlinear dynamics

## Abstract

Bulk metallic glasses are of critical interest for a wide range of applications, including their use in spacecraft gearboxes and mechanisms due to their excellent low-temperature, unlubricated wear resistance. Also of interest, is the potential for in-space manufacturing of metal alloys and the use of microgravity to determine fundamental thermophysical properties to inform ground-based modeling and experimentation. In this work, a Zr-based bulk metallic glass was processed in the electromagnetic levitator ISS-EML to determine undercooling, electrical resistivity, specific heat capacity, surface tension, and viscosity. A 6.5 mm sphere was vitrified during the processing, resulting in the first bulk metallic glass manufactured on board the international space station (ISS).

## Introduction

Metallic glasses are characterized by the absence of long range, periodic atomic arrangements, which leads to their special mechanical properties. The dislocation-based plasticity mechanisms observed in crystalline materials is absent in metallic glasses. This leads to large elastic strain limits and high yield strengths.^1^ This extraordinary combination of properties makes metallic glasses interesting for engineering applications, including low-temperature, unlubricated gears, and mechanisms for robotic spacecraft.^2,3^

A very stable and successful metallic glass developed by W. L. Johnson et al. was the glass-forming alloy Zr_41.2_Ti_13.8_Ni_10.0_Cu_12.5_Be_22.5_,^4^ referred to as Vit1 or LM1, which exhibits a critical cooling rate on the order of 1–10 K/s. Besides its good processability, the toxicity of Beryllium hindered some applications of this alloy and prevented in-space manufacturing due to astronaut toxicity concerns.

A Beryllium free alternative to Vit1 is the Zr_52.5_Cu_17.9_Ni_14.6_Al_10_Ti_5_, called Vit105 or LM105, which has been shown to have superior wear resistance compared to Vit1 in pin-on-disk testing, despite of lower hardness (474 Hv vs. 530 Hv).^3^ Its critical cooling rate has been reported to be about 10 K/s.^5^ Preliminary studies on thermodynamics of the alloy were already conducted using differential scanning calorimetry and electromagnetic levitation on board the space shuttle mission MSL-1.^6^

In this work, we processed the alloy LM105 in the electromagnetic levitator ISS-EML on board the international space station (ISS). The master alloy delivered by LiquidMetal Technologies, Inc. was research grade LM105 (<150 ppm oxygen). Afterwards, a spherical sample with diameter of 6.5 mm was suction casted in an arc-melting furnace. Special care was taken to reduce the possibility of oxygen uptake by evacuating the arc-melter to pressure below 5 × 10^−4^ mbar and flushing by high-purity Argon for several times. Afterwards, the arc-melter was filled by pure Argon (Ar 5.0, <2 vol.-ppm O_2_) for the melting process. Additionally, a Ti getter was molten in the arc-melting furnace before melting and suction casting of the LM105 sphere. The EML facility on board the ISS allows the contactless and containerless electromagnetic melting of spherical samples and the measurement of thermophysical properties in the liquid phase. Processing is possible in ultra-high vacuum or under an argon- or helium-atmosphere. The available gases are of high-purity (99.9999%).^7^

The sample can be positioned and heated by two independent electromagnetic fields generated by a coil system.^7–9^ Since the necessary strength of the positioner quadrupole field is rather low, it does typically not lead to substantial heating of the sample. Together with the wide range of heater dipole field strength that can be applied, the sample can be heated to a large range of temperatures (600–2000 K).

The ISS-EML was used for dual-purpose in the current work; (1) to measure the thermophysical properties of LM105 in a containerless environment to provide data for ground-based modeling and manufacturing, and (2) to demonstrate the possibility for in-space manufacturing of metal alloys with deep undercooling. Recently, space agencies such as NASA and ESA have invested heavily in the development of microgravity manufacturing equipment capable of fabricating metal parts on-orbit through additive manufacturing. The ISS-EML offers a unique manufacturing platform whereby metal alloys can be precisely heated, isothermally held, and undercooled to create bulk samples with known thermal processing history. In the current work, the platform was used to successfully vitrify a 6.5 mm diameter sphere of LM105 using radiative and conductive cooling in a helium atmosphere. This indicates that the achievable cooling rates and the environment are sufficient for the fabrication of high-quality metal samples. This could potentially augment the significant manufacturing efforts already being developed for the ISS. Such manufacturing facilities, making use of electromagnetic levitation and low or absent gravitational forces could also be of interest in long-term exploration missions or on an extraterrestrial base, such as on the moon.

## Results

**Fig. 1:**
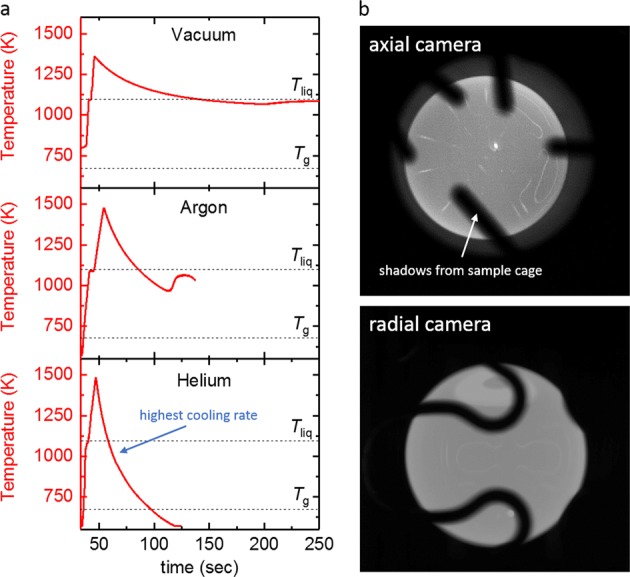
Containerless processing. **a** Temperature-time profiles of melt cycles performed under different atmospheres. The liquidus temperature *T*_liq_ = 1090 K is marked as well as the glass transition temperature *T*_g_ = 675 K **b** axial and radial camera image of the levitating, molten sample. The shadows on the samples stem from the (non-touching) sample cage

Typical melt cycles performed on the LM105 in different gas atmospheres are shown in Fig. 1a. As can be seen, the sample can easily be molten, overheated in the liquid phase and can afterwards cool down freely by radiative and conductive heat loss in the chosen processing environment. Figure 1a shows that in the cycle performed in helium atmosphere, the sample does not show recalescence, which means that the sample solidified as a glass. This shows evidence of the first bulk metallic glass sphere production in space.

Monitoring the sample during cooling with a small frame rate of 30 Hz allowed to trace the sample shape from the molten state, down to a temperature of about 700 °C, when the sample became invisible to the camera. The radius of the projection of a slightly distorted (elongated) sphere can be described as

1 $$R\left( \theta \right) = a_0 + a_2Y_2^0\left( {\cos \left( \theta \right),0} \right),$$

where $Y_2^0$ is the spherical harmonic for *l* = 2 and *m* = 0, *a*_0_ and *a*_2_ are coefficients. The relative elongation of the sample is described by *ε* *=* *a*_2_*/a*_0_. In every recorded frame, the sample shape was fitted with the aforementioned function in order to obtain the relative sample deformation. Once the heating field was turned off, the sample exhibited a residual elongation of *ε* *=* *a*_2_*/a*_0_ = 0.0081 ± 0.0005, which is equivalent to a distortion by only about 25 µm away from the perfect sphere of 6.5 mm diameter. This is equivalent to the ABMA ball grade 1000.

This shows that the absence of gravitational forces, and the small positioning forces necessary for stable levitation of the sample have negligible influence on the sample shape in the liquid phase, thus leading to a nearly perfect spherical sample. Hence, the sample can be levitated in the liquid phase while remaining a nearly perfect sphere, which may have future implications in the manufacturing of ball bearings. Figure 1b shows images of the sample in the liquid phase, taken from high-speed video recordings in the axial and radial direction, respectively. The shadows on the sample are due to the wires of the sample holder, which do not touch the sample, but are used to confine the sample during the absence of a positioning field before and after processing. The samples exhibited a clean surface in the solid and liquid phase and did not show any visible traces of oxide precipitates on the surface during the whole period of processing.

The containerless method allows processing of electrically conductive samples without contact to any foreign material. As such, heterogeneous nucleation often occurring due to the contact to container walls can hence be suppressed. The microgravity environment allows the decoupling of positioning and heating of electromagnetically levitated samples, which enables the melt to be largely undercooled.

When a liquid is cooled down, both atomic kinetics and the thermodynamic driving force influence the chance of the formation of crystalline nuclei. However, if the melt is cooled fast enough, thus slowing down atomic motions fast enough, it is possible to freeze the material into a glass. Viscosity is the macroscopic parameter that describes the inverse of the temperature dependent atomic mobility inside the liquid.

Hence, a critical cooling rate has to be achieved, in order to vitrify the melt. For production processes that aim at the manufacturing of bulk metallic glass parts, the challenge is to present reproducibly the conditions for the melt to cool down faster than the critical cooling rate. Predictions for this can be done by models that describe the mass transport inside the liquid and the heat transport inside and away from the melt. The heat capacity, being the inverse of the ratio of temperature change per energy change, is needed to predict the temperature change as a function of heat loss. Dependent on the manufacturing method, heat loss can take place e.g., by heat radiation (in vacuum). The heat loss per time of an object by heat radiation is given as

2 $$P_{\mathrm{{rad}}} = A\sigma _\mathrm{B}\varepsilon _{{\mathrm{tot}}}T^4$$

with the objects surface area, *A*, the Stephan-Boltzmann constant *σ*_B_ and the total hemispherical emissivity *ε*_tot_. Hence, precise values of the samples specific heat capacity, viscosity, as well as of its total hemispherical emissivity are needed as input parameters for models to develop and optimize metallic glass production. This is the case for traditional and new production technologies, such as casting, injection molding, or additive manufacturing.

### Electrical resistivity

By measurement of the sample’s impedance, the electrical resistivity and the sample diameter can be obtained as a function of temperature during the cooling phase. The details of the method are outlined in ref. ^10^ and also in the Methods section.

**Fig. 2:**
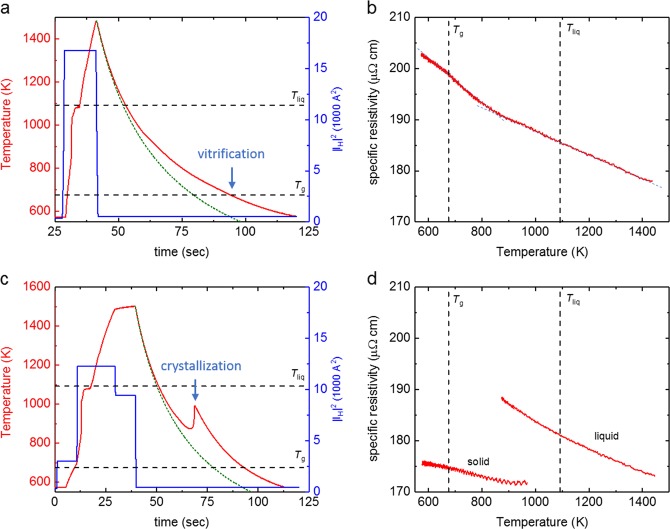
Vitrification and Crystallization. **a** Temperature-time profile of one cycle where the sample solidified as a glass, evident due to the absence of recalescence. The green dashed line presents the predicted cooling curve using a heat loss model with constant values for specific heat and emissivity **b** specific resistivity measured during the cooling phase in the cycle shown on the left. **c** Temperature-time profile of one cycle, in which the sample solidified by crystallization, together with a predicted cooling curve using a heat loss model with constant values for specific heat and emissivity. **d** The specific resistivity values which were measured alongside

As can be seen in Fig. 1a, under helium atmosphere, it was possible to achieve a cooling rate of about 8 K/s in average, which is faster than the critical cooling rate (~10 K/s^6^) for LM105. Figure 2c presents one case, where under similar conditions, the sample did not vitrify, but formed crystals.

Figure 2a, c shows two cycles that were performed in a 350 mbar He atmosphere, in order to measure the temperature dependent specific resistivity of the sample. In addition, the cooling curves in Fig. 2a, c were modelled by a heat loss model,^11^ using average values for the specific heat capacity and total hemispherical emissivity (see following section).

In both cases presented in Fig. 2, the electrical resistivity is increasing with decreasing temperature. This is often observed in disordered metals and the slope is in accordance with the expected slope, following the Mooij’s correlation.^12^

As can be seen in Fig. 2b, in case of vitrification, the electrical resistivity is changing without discontinuity, while in the case of crystallization (Fig. 2d), a sudden decrease in resistivity is recorded during crystallization. In Fig. 2b, the resistivity however shows a pronounced kink at about 850 K (dashed lines as a guide to the eye). This can be attributed to a change in electronic scattering due to structural changes, presumably a liquid-liquid transition, as was also observed at a temperature *T*/*T*_g_ ~ 1.2 in Zr_58.5_Cu_15.6_Ni_12.8_Al_10.3_Nb_2.8_^13^ and for Vit1,^14,15^ Vit106, LM7.^15^ Also for LM105, a decomposition on the nanometer scale was observed in time resolved SANS experiments.^16^ Such phase separation seems to be a general phenomenon appearing in strongly undercooled metallic liquids with good glass-forming ability.

### Specific heat capacity and hemispherical emissivity

**Fig. 3:**
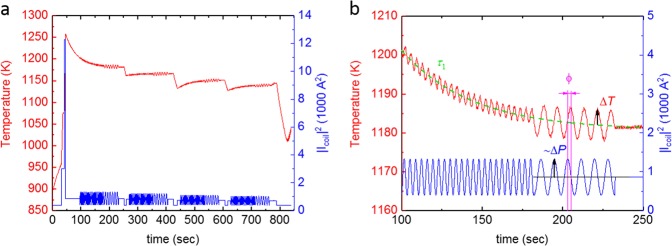
Modulation Calorimetry. **a** Typical modulation calorimetry cycle, performed under vacuum **b** details of the first modulations

The specific heat capacity and total hemispherical emissivity were determined by applying modulation calorimetry.^17–20^ Details of the modulation calorimetry method are presented in the Method section. The diagram in Fig. 3a shows a typical measurement cycle performed under vacuum. The left ordinate shows the temperature as a function of time, while the right ordinate shows the square of the heater current |*I*_coil_|^2^, which is proportional to the power dissipated in the sample. Figure 3b gives a detailed view on the first modulations of the cycle. The specific heat capacity can be obtained from the temperature oscillation Δ*T*, which follows the sinusoidal power modulation Δ*P* at a certain modulation frequency *f*_mod_.

**Fig. 4:**
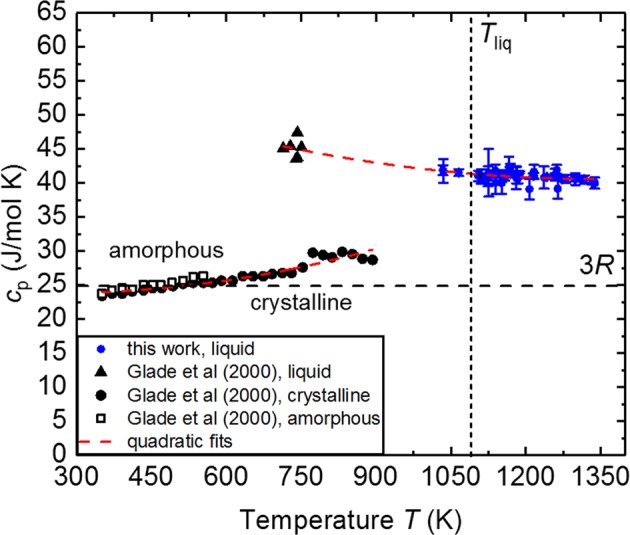
Specific Heat Capacity. **a** Specific heat capacity of LM105 in the liquid phase (this work), in the solid and deeply undercooled phase (Glade et al.^6^)

The results of the modulation calorimetry measurements are presented in Fig. 4. The gas atmosphere has no apparent effect on the determined specific heat capacity values. The molar specific heat capacity, shown in Fig. 4 changes from 40.1 J/mol·K at 1350 K in the stable liquid phase in a linear manner to 41.7 J/mol·K in the undercooled liquid region at 1050 K. The measurements are shown in combination with earlier investigation on the MSL-1 space shuttle flight mission^6^ in Fig. 4.

The phase shift ϕ between the power and temperature oscillation is a sensitive measure of the sample’s apparent thermal conductivity, a combination of thermal conductive transport and thermal transport by the fluid flow.^21^ The measured phase shift confirms that the measurement was performed in the adiabatic regime.

The total hemispherical emissivity was determined from the cycles performed in vacuum. The average value of *ε*_tot_ varies slightly between 0.26 at 1105 K and 0.27 at 1265 K. Taking an average value for the specific heat in the liquid phase and for the total hemispherical emissivity, one can model the cooling curves (see Fig. 2a, c) using a heat-loss model.^11^ A good agreement is found, which supports the accuracy of the measured values.

### Surface tension and viscosity

**Fig. 5:**
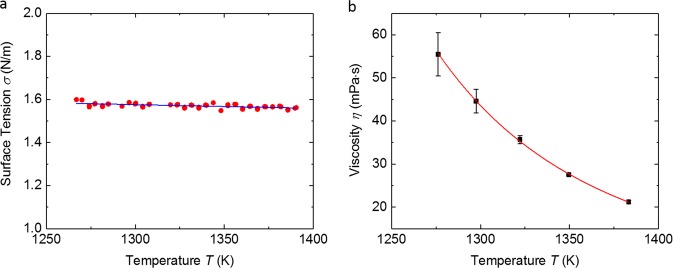
Surface Tension and Viscosity. **a** Surface tension as a function of temperature. **b** Viscosity as a function of temperature

The absence of external forces on the sample also eases the analysis of surface tension and viscosity using the oscillating drop method,^22^ due to the absence of frequency splits of the samples surface oscillations.^23^ The surface tension was measured in vacuum and derived from the samples surface oscillation frequency. The result is presented in Fig. 5a. A linear fit to the data allows the expression of the surface tensions temperature dependence as:

3 $$\sigma \left( T \right) = \left( {1.612 \pm 0.008} \right) - \left( {1.72 \pm 0.31} \right) \times 10^{ - 4} \times \left( {T - 1091\,\mathrm{K}} \right)$$

The oscillating drop method also allows for the analysis of the sample’s viscosity (details can be found in the Method section). The temperature dependent viscosity is shown in Fig. 5b and can be expressed by the Vogel-Fulcher-Tammann equation

4 $$\eta \left( T \right) = \eta _0{\mathrm{exp}}\left( {\frac{{DT_0}}{{T - T_0}}} \right)$$

with the parameters *η*_0_ = 0.12 mPa·s, *T*_0_ = 705.1 K, *D* = 5.0 in the temperature range of 1270–1390 K. Even though the given parameters describe the viscosity in the measured temperature range, the parameters may not be suitable to extract e.g., the materials fragility. The viscosity is higher than measurements of Mukherjee et al.^24^ performed in a ground-based electrostatic levitation facility. It has to be noted, that the LM105 sample investigated by Mukherjee et al. was crystalizing, for cooling rates of about 20 K/s, while we can confirm, in accordance with Lin et al.,^5^ a critical cooling rate of about 10 K/s. Hence, the higher viscosity found in^24^ could be an effect of a higher oxygen concentration.

The viscosity of LM105 measured in this work from 1270-1390 K and the data measured by Evenson et al. in a temperature range from 650–700 K^25^ can be described using the free volume model^26^

5 $$\log \left( \eta \right) = \frac{{A + 2B}}{{T - T_{0} + \sqrt {\left( {T - T_{0}} \right)^{2} + 4v_{\mathrm{a}}\zeta_{0}T} }}$$

with $B = v_\mathrm{m}\zeta _0{\mathrm{log}}(e)$. Here, *ν*_m_ is the molecular volume, *ν*_a_ and *ζ*_0_ are constants that describe the local free energy. Using the Kauzmann temperature *T*_K_ = 638 K^6^ as the characteristic temperature *T*_0_, we obtain: A = −4.72, *ν*_m_/*ν*_a_ = 213.86, and *ν*_a_*ζ*_0_ = 25.74.

## Discussion

We demonstrated the use of the ISS-EML to determine thermophysical properties of the bulk metallic glass former LM105 in the liquid state. This included the measurement of properties important for manufacturing, including undercooling in different gas atmospheres, surface tension, viscosity, specific heat, and total hemispheric emissivity. Furthermore, we successfully fabricated a 6.5 mm diameter glassy sphere under microgravity, cooled by radiative and conductive cooling in a helium atmosphere.

Lin et al. studied the time-temperature-transformation (TTT) diagram of LM105 with respect to the oxygen concentration in the sample and found a strong dependence of the critical cooling rate for vitrification on the oxygen concentration.^5^ He found an increasing critical cooling rate for an increasing oxygen content. The ISS-EML was demonstrated to be an excellent platform for avoiding contamination of the metallic glass sample, thus promoting glass formation in the spherical shape.

**Fig. 6:**
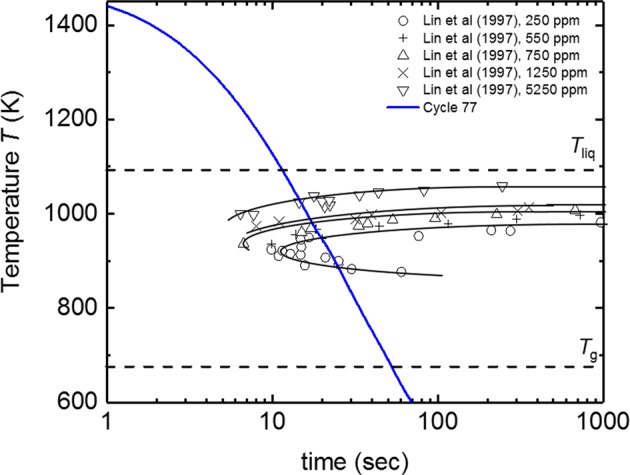
Temperature Time Transformation Diagram A typical cooling curve (cycle 77) during which the sample vitrified, together with ESL measurements of the TTT-diagram of LM105.^5^ The cooling curve (blue) passes through the TTT-diagram (even for the TTT curve measured on a sample with 250 ppm oxygen) without crystallization

A typical cooling curve is shown in Fig. 6 (cycle 77) in comparison with the TTT diagrams obtained by Lin et al.^5^ for different concentration of oxygen impurities in a ground-based electrostatic levitator. The cooling rate achieved by the radiative and conductive cooling in He atmosphere the sample should have crystallized based on the TTT diagram by Lin et al., if the oxygen concentration in the sample was higher than 250 ppm. Instead, the sample has sometimes vitrified during cooling under microgravity.

This is explained by a potentially cleaner sample compared to the samples studied by Lin et al.^5^ (initial oxygen concentration was better than 150 ppm), or by the lower level of fluid flux within the melt, taking place in our sample during processing in microgravity compared to the measurement conditions in a ground-based electrostatic levitator.

However, the possibility to vitrify the sample during undercooling and to have a strongly reduced fluid flow within the sample demonstrates the advantages of the microgravity processing on the ISS-EML compared to a ground-based levitation. Applying a surrogate model for convective flow inside electromagnetically levitated molten droplets,^27^ we can predict the Reynolds number in the droplet during cooling to be below *Re* = 15. Due to the relatively high viscosity, this is much smaller than for other metals processed under comparable process conditions.^27^

Thermophysical properties of LM105 (specific heat capacity, surface tension, viscosity, total hemispherical emissivity) in the stable and undercooled liquid phase were measured. Based on this, the design and optimization of traditional and new production methods in space and on ground, such as casting, laser melting, and laser-aided additive manufacturing, are now possible.

The experiments showed that the highest undercooling was provided by cooling in helium gas and that full vitrification of the 6.5 mm diameter sample could be detected through thermal imaging of the sample during cooling. This work demonstrated the dual-use of the ISS-EML as a platform for measuring critical properties of metallic alloys for use in ground-based manufacturing and it also demonstrated its potential use as a space-based manufacturing platform by successfully producing a macroscale spherical bulk metallic glass sphere; the first made in space. Future work will involve returning metallic glass spheres from orbit and studying microstructure, sphericity and mechanical properties for potential use as ball bearings.

## Methods

### Electromagnetic levitator on board the ISS

The electromagnetic levitation facility ‘TEMPUS’ (from german: Tiegelfreies Elektromagnetisches Prozessieren Unter Schwerelosigkeit) has a long history of numerous rocket missions, parabolic flights and two space shuttle missions on which it was flying to process metallic alloys under microgravity.^28^ Building on this expertise, since 2015, the international space station (ISS) facilitates the electromagnetic levitator ISS-EML in the European science module ‘Columbus’.^9^

The electromagnetic levitator ISS-EML consists of a process chamber, which is equipped with a coil system that allows the superposition of independent high frequency electromagnetic heating (370 kHz) and positioning (150 kHz) fields.^8^ This allows good visibility of the levitated sample for two high-speed cameras that are mounted axially and radially, in order to track the samples movements and oscillations. The axial camera also contains a pyrometer that enables the precise measurement of the sample temperature.

While not processed, the samples are kept in the sample chamber which is connected to the process chamber. The sample chamber can contain 18 different samples of about 6–8 mm diameter each. The samples are contained in sample “holders”, which is in this case a wire cage, which prevents the sample from floating away, when the positioning field is absent.

### Electrical resistivity

The electrical resistivity of the sample can be measured inductively during cooling using a dedicated measurement equipment for measurement of the impedance of the resonating circuit built by the induction coil and the parallel capacitor of the ISS-EML.^10^ By measuring the heater current, heater voltage and phase shift between them, the impedance of the resonant circuit can be determined. By performing an additional measurement without sample, the impedance caused by the sample (including the sample holder) can be calculated. Furthermore, the complex sample impedance can be approximated as^10^

6 $$Z_S\left( {\omega _{{\mathrm{Htr}}},a,\rho } \right) = C \cdot \omega _{{\mathrm{Htr}}} \cdot a^3\left[ {\frac{1}{q} - \frac{1}{{q^2}} + i\left( {\frac{1}{q} - \frac{2}{3}} \right)} \right]$$

with

7 $$q\left( {\omega _{{\mathrm{Htr}}},a,\rho } \right) = \frac{a}{\delta } = a \cdot \sqrt {\frac{{\mu _0\omega _{{\mathrm{Htr}}}}}{{2\rho }}}$$

where *a* is the sample radius and *ρ* the specific resistivity of the sample. The constant *C*, also called coil constant, depends on the coil geometry. The heaters angular frequency is denoted by *ω*_Htr_. The quantity *δ* represents the skin depth, with *µ*_0_ the magnetic permeability of the material. As derived by G. Lohöfer,^10^ the real and imaginary parts of the sample impedance can be used to obtain the sample radius *a*(*T*), and the samples resistivity *ρ*(*T*) when the coil constant is known. This coil constant, which describes the effect of the coil and sample geometry, as well as the effect of the sample cage, is obtained by the measurement of a solid Zr reference sample at to the α → β phase transition, where the electrical resistivity is known. Since the Zr sample is placed within the cage with exactly identical geometry, the influence of the cage on the measurement of the LM105 sample and the Zr sample as a reference will be the same.

### Modulation calorimetry

The modulation technique to study thermophysical properties, using a modulated power input to achieve a modulated temperature response was discovered by Corbino 1910^29^ and is nowadays the basis for a number of measurement methods to determine specific heat capacity and thermal conductivity.^17,20,30–32^ The certain AC modulation method for electromagnetic levitation, applied in this study was invented by Fecht et al.^17^ and later applied and refined by Wunderlich et al.^19,21,33–37^

The applied electromagnetic heater field dissipates the power *P*_H_ in the sample, which can be calculated by knowing the coil current |*I*_coil_|^2^ using

8 $$P_H = G_H \cdot \left| {I_{\mathrm{coil}}} \right|^2$$

where the coupling coefficient *G*_H_(*ρ*(*T*), *a*(*T*), *f*_H_) can be obtained using an analytical model,^38^ taking the coil geometry into account, as well as the samples temperature dependent radius *a*(*T*) and resistivity *ρ*(*T*).

However, the model used here^38^ is only strictly valid for a perfectly spherical sample. The applied heater field exerts a force on the sample, which can lead to a slight elongation of the droplet (when viewed from the radial camera). This is balanced by the droplets surface tension and the positioner field. A constant shape deformation can reduce the average projected diameter and the dissipated electrical power in the sample.

Calculations of Zhang et al.^39^ show, that for the factor $|a \cdot \varepsilon \sqrt {\omega _{{\mathrm{Htr}}} \cdot \mu \cdot \rho ^{ - 1}} | \ll 1$, the difference between dissipation in a perfect and distorted sphere are negligible.

The analysis of high-speed video recordings, taken during the ac calorimetry cycles at different average heating coil currents shows that the relative elongation of the sample is below *ε* = 2.5%. The factor $|a \cdot \varepsilon \sqrt {\omega _{{\mathrm{Htr}}} \cdot \mu \cdot \rho ^{ - 1}} |$ = 0.1 << 1. Hence, the effect of sample distortion can be neglected.

The power consumption of the cage wires has been simulated recently and is well below 2%, which allows us to neglect the power dissipation in the cage wires.

The temperature response to a sinusoidal power modulation *P*(*t*) = *P*_0_ + Δ*P* · sin(2·π·*f*_mod_ ·*t* *+* *φ*_0_) is given as *T*(*t*) = *T*_tr_(*t*) + *T*_0_ + Δ*T* · sin(2·π·*f*_mod_ ·*t* *+* *φ*_1_).

Figure 3a shows a typical measurement cycle, while Fig. 2b shows details of the first modulations of the cycle. It can be seen that the power is modulated sinusoidally, using two different frequencies at a constant average power. The temperature decay is characterized by the external relaxation time *τ*_1_. The temperature response lags behind the power signal by a phase angle *ϕ*. For each average temperature *T*_0_, the heat capacity *C*_p_ of the specimen can be obtained using,^17,20,21^

9 $$C_p = \frac{{\Delta P}}{{2 \cdot \pi \cdot f_{{\mathrm{mod}}} \cdot \Delta T}} \cdot f_c\left( {f_{{\mathrm{mod}}},\tau _1,\tau _2} \right)$$

where *f*_c_ represents a correction function, accounting for the effects of heat loss with the external relaxation time *τ*_1_ and the internal relaxation time *τ*_2_.

The internal relaxation time *τ*_2_ is characterizing the internal thermal conductance—the heated volume at the sample equator is indirectly heating the rest of the sample, due to heat transport carried by fluid flow and by thermal conduction of the liquid. The relative importance of fluid flow over thermal conductivity can be expressed by the Biot-number. In typical processing conditions, the Biot-number Bi < 0.01, and consequently, the internal relaxation time can be expressed using the thermal conductivity *k* and the sample radius *a* by^17^

10 $$\tau _2 = \frac{{3 \cdot C_p}}{{4 \cdot \pi ^3 \cdot k \cdot a}}$$

Under the same conditions of a small Biot-number, the phase shift *ϕ* = *φ*_1_ – *φ*_0_ between power and temperature modulation is given by

11 $${\mathrm{cos}}\left( \phi \right) = \left( {\frac{1}{{\tau _1}}\frac{1}{{\tau _2}} - \left( {2 \cdot \pi \cdot f_{{\mathrm{mod}}}} \right)^2} \right)\left( {\left( {\frac{1}{{\tau _1}}\frac{1}{{\tau _2}} - \left( {2 \cdot \pi \cdot f_{{\mathrm{mod}}}} \right)^2} \right)^2 + \left( {\frac{1}{{\tau _1}} + \frac{1}{{\tau _2}}} \right)^2\left( {2 \cdot \pi \cdot f_{{\mathrm{mod}}}} \right)^2} \right)^{ - 1/2}$$

The phase shift is a sensitive measure of thermal conductivity and heat loss. Phase angles *ϕ* above 90° express effects of the finite thermal conductivity, while phase angles *ϕ* below 90° point towards heat losses.

The external relaxation time *τ*_1_ depends on the atmosphere in which the experiment is conducted; however, in case of vacuum the external heat loss is only given by heat radiation and the relaxation time is given as^17^

12 $$\tau _1 = \frac{{C_p}}{{4 \cdot A \cdot \sigma_\mathrm{B} \cdot \varepsilon _{{\mathrm{tot}}} \cdot T^3}}$$

where *σ*_B_ is the Stefan-Boltzmann constant, *A* is the sample surface area and *ε*_tot_ is the total hemispherical emissivity.

### Oscillating drop method

**Fig. 7:**
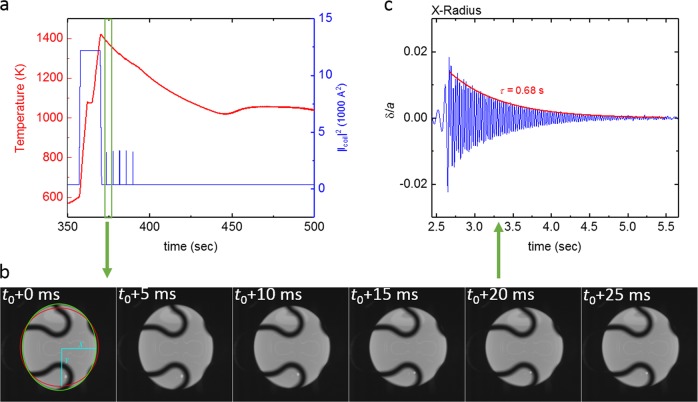
Oscillating Drop Method. **a** Typical cycle for measurement of surface tension and viscosity **b** after a heat pulse, surface oscillations are excited and detected by high-speed video cameras. **c** The surface oscillations are analyzed to determine surface oscillation frequency and decay time

The oscillating drop method,^22,40^ builds on the measurement of the samples surface oscillations. Figure 7a shows a typical cycle where the oscillating drop method was applied. The sample is heated, melted, overheated, and subsequently cools down. During cooling, surface oscillations are excited by application of a short heater pulse. The surface oscillations are recorded by the two high-speed video cameras. Figure 7b shows exemplary images recorded shortly after the first heater pulse. Using a dedicated software, the average sample diameter *a*, the time dependent deviation *δ*(*t*) from equilibrium diameter is determined. The relative oscillation amplitude *δ*(*t*)/*a* is shown in Fig. 7c.

Under the microgravity conditions on board the ISS, the sample exhibits surface oscillations with a single frequency only.^41^ Strong sample rotations,^42,43^ or large sample deformation amplitudes^44^ can shift the frequency. However, in this study no strong sample rotations were observed. Also, only small deformation amplitudes were analyzed (see e.g., Figure 7b).

The recorded sample oscillation amplitudes are evaluated using a Fourier transformation algorithm in order to obtain the oscillation frequency *f*_osc_ as a function of sample temperature.

Then the surface tension can be calculated from Rayleigh’s equation^45^

13 $$\sigma \left( T \right) = \frac{3}{8}\pi f_{osc}\left( T \right)^2M$$

with the known sample mass *M*. The internal friction of the liquid droplet will lead to an exponential decay of the surface oscillation amplitude (see Fig. 7c). The decay time constant *τ* can hence be used to determine the samples viscosity by applying Lambs equation^46^

14 $$\eta \left( T \right) = \frac{3}{{20\pi }}\frac{M}{a}\frac{1}{\tau }$$

However, both, the damping and surface oscillation are not independent phenomena, and hence, this equation is only valid for the case that the surface oscillation frequency is much higher than the inverse of the decay time (τ^-1^ « *f*_osc_).^47,48^

### Reporting summary

Further information on research design is available in the Nature Research Reporting Summary linked to this article.

## Supplementary information

Reporting Summary Checklist

## Data Availability

The datasets generated during and analyzed during the current study are available from the corresponding author on reasonable request.
